# Chronic Urticaria and Malignancy: A Review Uncovering the Common Links

**DOI:** 10.3390/biomedicines14010229

**Published:** 2026-01-21

**Authors:** Eralda Lekli, Mehmet Hoxha, Maria Bova, Juarda Gjonbrataj, Kleida Mati, Gentian Vyshka, Etleva Qirko

**Affiliations:** 1 Department of Internal Medicine, Faculty of Medicine, University of Medicine Tirana, 1005 Tirana, Albaniaj.gjonbrataj@gmail.com (J.G.);; 2 Salus Hospital Tirana, 1000 Tirana, Albania; 3 Biomedical and Experimental Department, Faculty of Medicine, University of Medicine Tirana, 1005 Tirana, Albania; 4 Service of Allergology, University Hospital Center Mother Teresa, 1005 Tirana, Albania; 5 Division of Internal Medicine 2, Department of Medicine and Medical Specialties, A. Cardarelli Hospital, 80131 Naples, Italy; bovamaria@virgilio.it; 6 Oncology Department, American Hospital, 1000 Tirana, Albania

**Keywords:** chronic urticaria, malignancy, mast cells, autoantibodies, IgE

## Abstract

**Background**: Chronic urticaria (CU) is a complex skin condition, frequently challenging both patients and clinicians and requiring wise individualized management. While allergy, autoimmunity, and depression are recognized comorbidities, evidence linking CU and malignancy remains underexplored. Screening for malignancy is not a routine standard of care for CU patients. **Methods**: A literature review was conducted to explore the potential risk or associations, including immune mechanisms, between CU and malignancy, based on searches in the PubMed and Google Scholar databases. **Results**: Scientific evidence on the malignancy risk in CU and its causal relationship is limited to a few population-based studies and case reports. A higher incidence of hematologic malignancy in CU patients has been reported in several publications, but the overall risk of malignancy in the CU population remains controversial. Antihistamine resistance, ultra-low IgE, and older age at the time of CU diagnosis may be related to a higher risk of malignancy, especially shortly after CU diagnosis. Immunological pathways linking CU and cancer are not clear. Immune system dysregulation, including alterations in immune checkpoints, is a feature of both cancer and CU. Such dysregulation may promote immunotolerance, abnormal immune responses, and mast cell activation through novel autoantigens and autoantibodies involved in the tumor microenvironment. **Conclusions**: There is growing, although limited, evidence suggesting a possible link between CU and malignancy, especially hematologic cancers. Large multicenter cohort studies are warranted to determine whether CU may act as a clinical harbinger of malignancy and to identify patient subsets that may benefit from targeted cancer screening.

## 1. Introduction

The possible connection between chronic urticaria (CU) and cancer is an evolving and complex area of research, with growing interest in analyzing the common risk factors and understanding how mast cell (MC) activation may serve as a potential link.

Chronic urticaria is defined as the presence of wheals, angioedema, or both lasting longer than 6 weeks. It may have symptoms that occur daily or nearly every day and follow an intermittent or recurring pattern. The signals that activate MC in urticaria are heterogeneous and include T cell-driven cytokines and autoantibodies [1]. In chronic spontaneous urticaria (CSU), types I and II autoimmune responses play a key role. These include IgE antibodies targeting self-antigens and IgG autoantibodies directed against IgE or its receptors, which lead to MC activation [2].

While there is evidence suggesting a connection between chronic urticaria and an increased risk of malignancies [3,4], several case reports have additionally linked CU to specific cancers. However, the link remains controversial, and the design of studies in this area varies considerably. There is limited direct research exploring the link between MC-mediated inflammation, low IgE levels in autoimmune urticaria, and the risk of malignancy. Cancer cell antigens may act as autoantigens interfering with the CU physiopathology. Available empirical data have not demonstrated definitive mechanisms underlying this potential association.

Individuals with autoimmune disorders frequently exhibit abnormal immune activity, which may impair the recognition and elimination of cancer cells from the organism. Immune checkpoint receptors, such as program death-1 (PD-1) and T-cell immunoglobulin and mucin domain 3 (TIM-3), have been studied in autoimmune disease [5,6], allergic disease [7], and malignancy [8]. Dysregulation or malfunction of TIM-3 has been connected to the exacerbation of autoimmune diseases in various preclinical models [9,10]. TIM-3 plays a significant role in modulating both innate and adaptive immunological responses by acting as a negative regulator of immune cells. It promotes immunological tolerance by inducing T cells’ apoptosis or impeding the activation of innate immune cells. PD-1 expression is upregulated following the activation of T cells. As highlighted in the cited studies, tumors use the PD-1-mediated inhibitory pathway to reduce anti-tumor immunity and avoid immune system destruction, which increases tumor survival and growth [8,11,12].

A recent study demonstrated a significantly higher expression of PD-1 in CSU patients, an insignificantly decreased expression of TIM-3 compared with the healthy group, and a positive correlation between the expression of PD-1 and TGF- β molecules and disease activity. The higher expression of PD-1 molecules and IL-10 was associated with the disease severity, suggesting that the immune system is trying to control inflammation and reduce the disease severity [6].

## 2. Materials and Methods

This literature review is based on English-language articles, mainly open access, published in the PubMed and Google Scholar databases. The main literature search keywords used were “Chronic Urticaria” and “malignancy” or “cancer”. Additionally, a literature search was performed to address MCs’, autoantibodies’, autoantigens’, and IgE’s role in both chronic urticaria, malignancy, and their comorbidity. The investigation of the common physiopathology of malignancy and CU focused on the immune mechanisms and recent concepts in CSU. Articles reporting a risk or association of malignancy in patients with chronic urticaria, case reports, and literature reviews were included. Individual case reports of CU and malignancy association were included if published after 2018. Case reports published before 2018 were extracted from the 2018 systematic case collection publication [13]. Reference lists of the selected papers were also manually reviewed to identify additional relevant articles. Papers that could not be fully accessed were included if their abstracts contained relevant information. To avoid overlapping information, the earliest published paper was cited. The search was finalized at the end of June 2025.

The findings from the literature review were organized into two foci. First, we summarize the reported risk findings regarding the incidence and co-incidence between chronic urticaria and malignancy from various studies and case reports. Second, we explore potential shared immunopathological mechanisms underlying both conditions. Brief remarks derived from the reviewed literature are included when relevant.

## 3. Results

### 3.1. Reported Frequency and Risk of Malignancy in CU Patients

According to several studies, chronic urticaria has been associated with an increased risk of malignancy, particularly hematologic cancers. A recent Danish population-based cohort reported the risk of any cancer to be 0.7% (95% CI 0.6–0.7) during the first year of follow-up after urticaria diagnosis. In same study, an overall standardized incidence ratio (SIR) of malignancy was reported as 1.09 (95% CI 1.06–1.11) and an SIR (1.49, 95% CI 1.38–1.62) during the first year after urticaria diagnosis. After the first year, the increased risk remained at 6% (SIR 1.06, 95% CI 1.04–1.09) [3]. The study showed a modest increase in cancer risk among individuals with urticaria, but the absolute risk remained low [3]. A Taiwanese population-based cohort study in 12,720 CU cases found that patients with CU had an SIR of 2.2 for cancer, with hematologic malignancies exhibiting an SIR of 4.1. The risk was notably higher among individuals aged 20 to 39 years, and most cancers were detected within the first year of CU diagnosis [4]. Two other studies did not find an increased risk of CU for malignancies [14,15]. An Italian study reported a risk of 0.007% from data covering a large cohort of 1493 consecutive CU patients and confirmed the rarity of the association [14]. A Swedish study concluded that chronic urticaria was not statistically associated with malignancy in general. It reported a diagnosed malignancy in 36 of 1155 patients with chronic urticaria, while the expected number of malignancies was 41. In 23 patients, the malignancy appeared during the same year as the onset of urticaria or later, while the expected number of malignancies in this period was 25.6 [15].

### 3.2. Cancer-Associated CU and CU–Malignancies Comorbidity

In the literature, four features have been proposed to characterize cancer-associated CSU: (i) antihistamine resistance, (ii) onset before malignancy is diagnosed (mostly 2–8 months), (iii) resolution after effective treatment of cancer, and (iv) recurrence upon relapse of cancer [14,16]. In all histamine-resistant CU patients, a careful medical history should be taken, as cutaneous disease may occasionally be the sole manifestation of an underlying malignancy [4].

A higher incidence of hematologic malignancy in CU patients was reported in several studies (SIR 4.1; 3.1–5.4), with the highest risk of non-Hodgkin lymphoma (SIR, 4.4; 95% CI, 3.0–6.1), as reported by Chen et al. [4]. In a recent study, Sorensen et al. reported data for non-Hodgkin lymphoma (SIR 2.91, 95% CI 1.92–4.23) in urticaria patients [3].

Another study found patients with CU and Monoclonal Gammopathy of Undetermined Significance (MGUS) had a higher rate of hematologic malignancy association, at 15% compared with 0.9% of CU patients without MGUS [16]. Hematological malignancies were reported in eight patients (25%), among 32 case reports of malignancy-related CU found in the literature. Among 797 patients with CU, malignancy was found in 142 cases. Patients presenting with a new diagnosis of CU at an older age (>56 years) were more likely to have associated underlying MGUS [17]. Out of 180 patients with CU, 6 (3.3%) had malignancies [18]; out of 181 patients aged 65 and older, 23 (13.8%) had malignancies [19]; and out of 108 patients, 4 (3.7%) had malignancies as a comorbidity [20]. Another study reported that neoplastic disease was associated with chronic urticaria in only 2 patients out of the 348 evaluated [21]. A summary of these studies and cases reports is presented in Table 1.

### 3.3. Chronic Urticaria and Malignancy: The Role of Autoantibodies

Autoantibodies play crucial roles in the etiology and pathobiology of various groups of diseases, including autoinflammatory conditions and cancer. While antibodies targeting specific self-antigens are often indicative of ongoing or developing pathological processes, detectable autoantibodies can also exhibit protective effects, which may be beneficial in certain pathophysiological conditions [30]. Autoantibodies in chronic urticaria and cancer may arise from the breakdown of immune tolerance and dysregulated B-cell activity.

Chronic spontaneous urticaria is considered to be an autoimmune disease. Pathogenesis in “autoallergic CSU” or autoimmunity type I is mediated by IgE autoantibodies to self-antigens and, in autoimmunity type IIb, with MC-directed activating autoantibodies [2]. The literature proposes possible mechanisms to explain the link between urticaria and cancer. One possibility is that the tumor disrupts normal immune function or produces substances that activate MC, leading to the development of urticaria. Alternatively, chronic urticaria itself might alter the immune system over time, impairing the body’s ability to detect and eliminate cancer cells [16].

The incidence of cancer has been reported to increase in many patients with autoimmune diseases [31]. Although a connection might exist, it is still unclear whether underlying autoimmunity leads to cancer, with inflammation promoting malignancy, or whether immune responses targeting tumor antigens are responsible for the development of autoimmune conditions [32].

Gene polymorphism may lead to impaired regulation or a lowered threshold for lymphocyte activation, while environmental triggers such as infections or injuries can initiate or enhance the activation of self-reactive lymphocytes that have evaded normal control and can target autoantigens [33,34]. Autoantigens and autoantibodies are increasingly identified in a range of cancers, even when there is no evident underlying autoimmune disease. Autoantibodies have been detected against mutated proteins (neoantigens) and normal proteins that are overexpressed on cancer cells or released by cancer cells. The molecular function of such autoantibodies has been clarified only in certain cases [34].

Peripheral tolerance involving self-reactive lymphocytes T and B can be broken by self-antigen overexpressed in a tissue or if neoantigens, including mutated peptide epitopes, aberrantly spliced or aberrantly post-translationally-modified epitopes, or new discontinuous epitopes resulting from misfolding of the antigen, are presented to the host immune system [32,35]. It is supposed that cancer can be promoted or inhibited by the direct contribution of existing autoantibodies, but they may also simply serve as indirect markers of underlying immune processes that significantly influence cancer development [34].

Autoantibody-mediated immune dysregulation may be seen as a link between CU and malignancy pathogenesis. In autoimmune CU, autoantibodies trigger mast cell and basophil activation by targeting IgE or its receptor, leading to persistent histamine release and chronic urticarial symptoms. Autoantibodies in cancer may have dual roles: they may exert anti-tumor effects or support tumor progression through diverse pathways, such as interfering with immune checkpoints, promoting immune exhaustion and tolerance.

### 3.4. Chronic Urticaria and Malignancy: The Role of Mast Cells

CSU is considered an MC-driven skin disease with diverse activating signals [1,2], where MCs are pathologically hyper-responsive. When activated, they release mediators leading to vasodilation, plasma leakage, wheal formation, itch, and persistent symptoms when activation is sustained.

Elevated numbers of MCs are observed in both affected and unaffected skin areas in CSU, and their responsiveness is heightened during active urticaria, due to local inflammatory cytokines and neuropeptides. These MC-derived cytokines and neuropeptides, especially nerve growth factor, promote a Th2-type inflammatory response, which is particularly pronounced at the sites of wheal formation [36]. In CSU, the activation of dermal MCs to release histamine appears critical to the development of symptoms [2].

Additionally, histamine induces tumor cell proliferation by acting on tumor surface expressed H1 receptors [37,38]. In several studies, increased MCs at solid tumor sites have been noted, in keeping with their localization at sites of blood vessel development [39,40]. The ability of MCs to promote angiogenesis is viewed as a key process in promoting tumor development, according to the published research [41,42,43] Chronic low-grade inflammation is seen as a characteristic feature of malignancy, with mast cells constituting important components of the tumor-associated inflammatory microenvironment.

It is proposed that cancer may be linked to MC activation and CSU in two possible ways [16]: first, through the production and release of signals from the tumor or nearby tissue that attract and activate MCs [44]; second, by the tumor-derived antigens that are recognized by IgE antibodies, aligning with the concept of AllergoOncology [45].

MCs accumulate into the tumor microenvironment (TME) with the help of tumor cell-released chemoattractants [46]. Their functional role may be variable, according to the tumor type and local microenvironmental factors, whereby mast cells may facilitate tumor progression, exert inhibitory effects, or display a neutral influence on tumor behavior. MCs are known for releasing various proinflammatory substances, but they also play a role in immune suppression by producing immunosuppressive cytokines [38]. Once MCs infiltrate the tumor stroma, they contribute to the expansion and activation of regulatory T cells (Tregs), fostering an immunosuppressive environment that facilitates tumor growth [47]. MCs release numerous mediators that drive the formation of new blood vessels and degrade the surrounding extracellular matrix, directly supporting tumor growth [38].

Depending on the tumor type, MCs can promote an immunosuppressive environment through the release of IL-10, histamine, and TNF-α. They may also hinder the function of T cells and natural killer (NK) cells by releasing adenosine into the surrounding tumor environment [48]. Although not yet confirmed, tumor-derived mediators may potentially lower mast cell activation thresholds and induce systemic mast cell priming, leading to mast cell activation beyond the tumor site, including in the skin, where it manifests as chronic urticaria. CU as a mast-cell-driven paraneoplastic phenomenon is rare; however, it is biologically plausible because not all tumors recruit mast cells strongly, systemic mast cell responsiveness varies among individuals, and genetic and microenvironmental factors may influence mast cell sensitivity.

### 3.5. Chronic Urticaria and Malignancy: The Role of Total IgE

Over the past decade, it has become clear that immunoglobulin E (IgE) and cells expressing its high-affinity receptor FcεRI, traditionally associated with allergic responses, also contribute to the development and progression of multiple autoimmune disorders [49].

Elevated total IgE levels may indicate more active disease, longer disease duration, type I autoallergic CSU, a higher likelihood of responding to omalizumab, a faster relapse after stopping omalizumab, and a reduced response to cyclosporine. Conversely, low total IgE levels may suggest type IIb autoimmune CSU, a lower probability of benefiting from omalizumab, and a higher potential for a positive response to cyclosporine [50]. Therefore, measuring the total IgE is a valuable diagnostic tool and should be routinely included in the evaluation of CSU patients.

More than 200 autoantigens that are recognized by IgE have been found in CSU patients. IgE-anti-IL-24 was detected in all patients with CSU. In addition, the levels of IgE-anti-IL-24 in the blood were linked to the activity of CSU [51].

It seems that higher IgE levels are inversely associated with certain cancers, possibly reflecting a more active immune surveillance system. Conversely, very low IgE, as may be seen in autoimmune type IIb CSU, may correlate with an altered immune balance, which could indirectly facilitate tumor immune evasion.

IgE autoantibodies directed against tumor antigens have been identified both locally in tumor tissue and in the systemic circulation, indicating they may have a tumor-inhibiting function. Cross-reactivity between autoallergens needs to be investigated in future research [52].

It is supposed that tumors may express neoantigens, proteins that are either newly formed or abnormal, provoking the immune system to produce IgE antibodies against them. These antibodies bind to high-affinity FcεRI receptors on mast cells and basophils, leading to their activation both within the tumor microenvironment and systemically, a mechanism that has been investigated in experimental IgE-based immunotherapies to mediate antibody-dependent tumor cytotoxicity.

The evidence indicates that IgE antibodies may contribute to localized and durable anti-tumor protection, potentially involving CD23 and its Fc receptors in monitoring and controlling tumor growth in some of these mechanisms. It also raises the possibility that even the low circulating levels of IgE found in healthy individuals might support immune surveillance against tumors [53].

**Figure 1:**
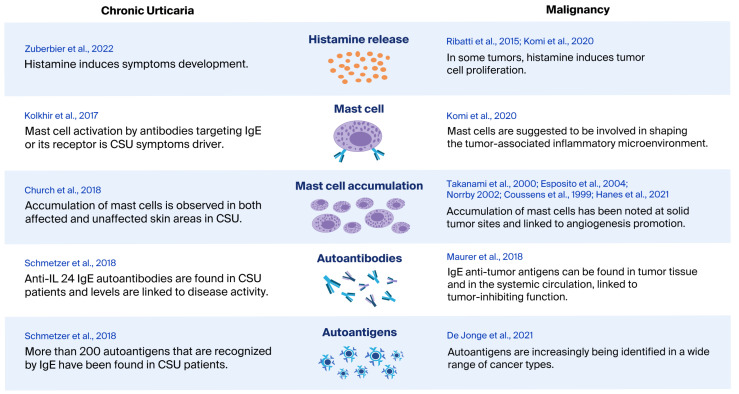
Possible common links in the immunopathology of CU and malignancy. Histamine release in CU is a symptom inducer [1]; in malignancy, histamine release may contribute to tumor proliferation [38]. Mast cell accumulation in CU skin (lesional and non-lesional) [36] and MC activation is a symptom driver [2]; mast cell accumulation in some tumor sites is linked to inflammation and angiogenesis [39,40,41,42,43]. IgE autoantibodies against self-antigens in CU lead to mast cell activation [2]; in cancer, IgE antibodies recognize tumor-derived antigens and have been linked to tumor-inhibitory functions [52]. Numerous autoantigens have been identified in chronic spontaneous urticaria [51], with an increasing number also being recognized in cancer [34].

High levels of IgE in the blood are usually linked to allergies or atopic diseases, but very low or no IgE might make it more difficult for the body to detect and fight tumors. This suggests that having the proper balance of IgE-related immune responses is important. Extremely low IgE levels could also be an unexpected marker that someone might be at higher risk of cancer [45], which may indicate that individuals with low IgE titers may benefit from increased cancer screenings. In Figure 1, we present potential common immune-mediated pathways between CU and malignancy.

## 4. Discussion

CU and malignancies may be linked in some patients, as suggested by case reports, in which urticaria remission follows cancer treatment or the recurrence of urticaria signals cancer relapse. Considering the publication bias related to the reporting of these two associated conditions and chance co-occurrence, these reports show limited evidence of an association. Studies that found a higher standardized incidence rate of malignancy among chronic urticaria patients included a higher number of cases, consisting in population-based cohorts, making the observation more reliable by providing stronger evidence. The available data indicated a modest increase in cancer risk among individuals with urticaria, while the absolute risk remained low [3]. It is suggested that an elevated short-term cancer risk may be partly due to increased diagnostic efforts following urticaria diagnosis. The availability of testing for malignancy and CU-related broader examinations and follow-ups, may be an important factor in the malignancy diagnosis rate in the published studies.

It is also possible that undiagnosed cancers could manifest as urticaria, or that both conditions share common risk factors.

The risk and association between cancer and CU, as well as the direction of this relationship, remain difficult to evaluate due to potential cofactors and confounders, including comorbidities (particularly autoimmune conditions and infections), medications, and malignancy prognosis.

However, higher cancer rates reported in the first-year post CU diagnosis, may serve as a confirmation of cancer-associated CU, taking into consideration the literature reporting the diagnosis of malignancy between 2 and 8 months after CU onset [14,16]. Studies have shown that advanced age at the time of the urticaria diagnosis may be linked to a higher likelihood of cancer comorbidity [17,20]. Broad high-cost screening approaches for the causes of urticaria are discouraged by the current guidelines, as the distribution of underlying etiologies appears to differ across regions worldwide, an aspect that is still poorly understood [1].

Although autoantibodies are primarily pathogenic and symptom-driving in chronic urticaria, in cancer they exert dual and context-dependent effects that shape tumor immunity and clinical outcomes.

The immune disbalance in CU may predispose to, result from, or coexist with tumor-associated immune dysregulation by sustaining chronic inflammation, altering immune cell function, and impairing anti-tumor immune surveillance.

Probably low IgE in chronic urticaria, type IIb autoimmune CSU, and antihistamine resistance may be a signal for malignancy screening, while the local effect of MCs in relation to the skin malignancy risk still needs to be investigated.

## 5. Conclusions

Many of the evaluated studies were observational and retrospective, which limits the ability to establish causality and confounding factors such as autoimmune diseases, medications, and comorbidities, thus complicating the results. The emerging research suggests that tumor-derived signals and immune dysregulation could promote MC accumulation and activation not only in CU but also in cancerous tissues, contributing to both inflammation and tumor progression.

While no definitive causal link has been established, immune checkpoint dysfunction in chronic urticaria may hypothetically impair tumor surveillance, particularly in autoimmune CU patients. This suggests the need for long-term surveillance and deeper research into cancer risk stratification in CU cohorts.

Larger well-designed multicentric prospective studies with long follow-up periods are needed to more accurately assess the potential risks of cancer in CU patients and better understand the underlying mechanisms. The current body of evidence is insufficient to recommend routine cancer screening for all patients with CU, but ongoing research is critical to clarifying these potential associations.

## Figures and Tables

**Table 1:** Summary of studies and cases reporting the risk of malignancy, CU-related malignancy, and CU–malignancy comorbidity.

Publication (Year)	Publication Type	Country	Population	Age	Number of Patients Included	Type of Reported Malignancy	Frequency of Reported Malignancy
Sørensen et al. (2024) [3]	Population-based cohort study	Denmark	Urticaria	All ages	87,507	All types of malignancy	0.7% for the first-year, standardized incidence rate (SIR) 1.49, after first year (SIR 1.06)
Rutecka et al. (2024) [22]	Case report	Poland	Chronic urticaria	61	1	Gastric cancer	NA
Royal-Preyra et al. (2024) [23]	Case report	Canada	Chronic urticaria	43	1	Breast cancer	NA
Sutton et al. (2024) [24]	Case report	Australia	Chronic urticaria	Pediatric	1	Chronic myeloid leukemia	NA
Khaliliya et al. (2023) [19]	Retrospective	Israel	Chronic urticaria	>65	181	Malignancy as comorbidity	13.8%
Junior et al. (2024) [18]	Cross-sectional	Brazil	Chronic urticaria	13–81	180	Cervical, bladder, breast, and colon cancer and Hodgkin’s lymphoma	6/180
Jiménez et al. (2022) [25]	Case report	Colombia	Chronic urticaria	25	1	Lung cancer	NA
Tracy et al. (2022) [26]	Case report	USA	Chronic urticaria	49	1	Chronic myelogenous leukemia	NA
Bizjak et al. (2021) [27]	Prospective cohort study	Slovenia	Cold-induced urticaria	18–73	35	Malignancy	1/35
Lugović-Mihić, L. et al. (2021) [28]	Retrospective	Croatia	Chronic urticaria	67	1	Prostate cancer	NA
Santiago-Vázquez (2019) [29]	Case report	Puerto Rico	Chronic urticaria	52	1	Colon adenocarcinoma	NA
Napolitano et al. (2018) [14]	Retrospective	Italy	Chronic urticaria	48.3 ± 18.1 years)	1493	1 lung cancer	0.007%
Larenas-Linnemann et al. (2018) [13]	Systematic collection of cases	NA	Urticaria	10–70	26	6 thyroid 4 lung 4 leukemia 4 GI 2 breast 1 IgA myeloma 1 Hodgkin’s lymphoma 1 prostate 1 ovarian 1 seminoma 1 astrocytoma	NA
Chen et al. (2012) [4]	Retrospective population-based cohort study	Taiwan	Chronic urticaria	All ages	12,720	All types of malignancy	SIR 2.2
Pigatto et al. (2000) [21]	Consecutive cases	Italy	Chronic urticaria	24–59	348	All types of malignancy	2/348
Karakelides et al. (2006) [17]	Retrospective	USA	Chronic urticaria	All ages	797	All types of malignancy Monoclonal gammopathy of unknown significance	142/797
Lindelöf et al. (1990) [15]	Retrospective	Sweden	Chronic urticaria	All ages	1155	All types of malignancy	36/1155

## Data Availability

No new data were created or analyzed in this study. Data sharing is not applicable to this article.
